# Essential *versus* accessory aspects of cell death: recommendations of the NCCD 2015

**DOI:** 10.1038/cdd.2014.137

**Published:** 2014-09-19

**Authors:** L Galluzzi, J M Bravo-San Pedro, I Vitale, S A Aaronson, J M Abrams, D Adam, E S Alnemri, L Altucci, D Andrews, M Annicchiarico-Petruzzelli, E H Baehrecke, N G Bazan, M J Bertrand, K Bianchi, M V Blagosklonny, K Blomgren, C Borner, D E Bredesen, C Brenner, M Campanella, E Candi, F Cecconi, F K Chan, N S Chandel, E H Cheng, J E Chipuk, J A Cidlowski, A Ciechanover, T M Dawson, V L Dawson, V De Laurenzi, R De Maria, K-M Debatin, N Di Daniele, V M Dixit, B D Dynlacht, W S El-Deiry, G M Fimia, R A Flavell, S Fulda, C Garrido, M-L Gougeon, D R Green, H Gronemeyer, G Hajnoczky, J M Hardwick, M O Hengartner, H Ichijo, B Joseph, P J Jost, T Kaufmann, O Kepp, D J Klionsky, R A Knight, S Kumar, J J Lemasters, B Levine, A Linkermann, S A Lipton, R A Lockshin, C López-Otín, E Lugli, F Madeo, W Malorni, J-C Marine, S J Martin, J-C Martinou, J P Medema, P Meier, S Melino, N Mizushima, U Moll, C Muñoz-Pinedo, G Nuñez, A Oberst, T Panaretakis, J M Penninger, M E Peter, M Piacentini, P Pinton, J H Prehn, H Puthalakath, G A Rabinovich, K S Ravichandran, R Rizzuto, C M Rodrigues, D C Rubinsztein, T Rudel, Y Shi, H-U Simon, B R Stockwell, G Szabadkai, S W Tait, H L Tang, N Tavernarakis, Y Tsujimoto, T Vanden Berghe, P Vandenabeele, A Villunger, E F Wagner, H Walczak, E White, W G Wood, J Yuan, Z Zakeri, B Zhivotovsky, G Melino, G Kroemer

**Affiliations:** 1 Gustave Roussy Cancer Center, Villejuif, France; 2 Equipe 11 labellisée par la Ligue Nationale contre le Cancer, Centre de Recherche des Cordeliers, Paris, France; 3 Université Paris Descartes/Paris V, Sorbonne Paris Cité, Paris, France; 4 INSERM, U1138, Gustave Roussy, Paris, France; 5 Regina Elena National Cancer Institute, Rome, Italy; 6 Department of Oncological Sciences, The Tisch Cancer Institute, Ichan School of Medicine at Mount Sinai, New York, NY, USA; 7 Department of Cell Biology, UT Southwestern Medical Center, Dallas, TX, USA; 8 Institute of Immunology, Christian-Albrechts University, Kiel, Germany; 9 Department of Biochemistry and Molecular Biology, Thomas Jefferson University, Philadelphia, PA, USA; 10 Dipartimento di Biochimica, Biofisica e Patologia Generale, Seconda Università degli Studi di Napoli, Napoli, Italy; 11 Department of Biochemistry and Medical Biophysics, University of Toronto, Toronto, ON, Canada; 12 Biochemistry Laboratory, Istituto Dermopatico dell'Immacolata – Istituto Ricovero Cura Carattere Scientifico (IDI-IRCCS), Rome, Italy; 13 Department of Cancer Biology, University of Massachusetts Medical School, Worcester, MA, USA; 14 Neuroscience Center of Excellence, School of Medicine, New Orleans, LA, USA; 15 VIB Inflammation Research Center, Ghent, Belgium; 16 Department of Biomedical Molecular Biology, Ghent University, Ghent, Belgium; 17 Barts Cancer Institute, Cancer Research UK Centre of Excellence, London, UK; 18 Queen Mary University of London, John Vane Science Centre, London, UK; 19 Department of Cell Stress Biology, Roswell Park Cancer Institute, Buffalo, NY, USA; 20 Karolinska University Hospital, Karolinska Institute, Stockholm, Sweden; 21 Institute of Molecular Medicine and Spemann Graduate School of Biology and Medicine, Albert-Ludwigs University, Freiburg, Germany; 22 Buck Institute for Research on Aging, Novato, CA, USA; 23 Department of Neurology, University of California, San Francisco (UCSF), San Francisco, CA, USA; 24 INSERM, UMRS769, Châtenay Malabry, France; 25 LabEx LERMIT, Châtenay Malabry, France; 26 Université Paris Sud/Paris XI, Orsay, France; 27 Department of Comparative Biomedical Sciences and Consortium for Mitochondrial Research, University College London (UCL), London, UK; 28 Department of Experimental Medicine and Surgery, University of Rome Tor Vergata, Rome, Italy; 29 Laboratory of Molecular Neuroembryology, IRCCS Fondazione Santa Lucia, Rome, Italy; 30 Department of Biology, University of Rome Tor Vergata; Rome, Italy; 31 Unit of Cell Stress and Survival, Danish Cancer Society Research Center, Copenhagen, Denmark; 32 Department of Pathology, University of Massachusetts Medical School, Worcester, MA, USA; 33 Department of Medicine, Feinberg School of Medicine, Northwestern University, Chicago, IL, USA; 34 Human Oncology and Pathogenesis Program and Department of Pathology, Memorial Sloan Kettering Cancer Center (MSKCC), New York, NY, USA; 35 Laboratory of Signal Transduction, National Institute of Environmental Health Sciences (NIEHS), National Institute of Health (NIH), North Carolina, NC, USA; 36 Tumor and Vascular Biology Research Center, The Rappaport Faculty of Medicine and Research Institute, Technion Israel Institute of Technology, Haifa, Israel; 37 Neuroregeneration and Stem Cell Programs, Institute for Cell Engineering (ICE), Departments of Neurology, Pharmacology and Molecular Sciences, Solomon H Snyder Department of Neuroscience, Johns Hopkins University School of Medicine, Baltimore, MD, USA; 38 Adrienne Helis Malvin Medical Research Foundation, New Orleans, LA, USA; 39 Department of Experimental and Clinical Sciences, Gabriele d'Annunzio University, Chieti, Italy; 40 Department of Pediatrics and Adolescent Medicine, Ulm University Medical Center, Ulm, Germany; 41 Department of Systems Medicine, University of Rome Tor Vergata, Rome, Italy; 42 Department of Physiological Chemistry, Genentech, South San Francisco, CA, USA; 43 Department of Pathology and Cancer Institute, Smilow Research Center, New York University School of Medicine, New York, NY, USA; 44 Laboratory of Translational Oncology and Experimental Cancer Therapeutics, Department of Medicine (Hematology/Oncology), Penn State Hershey Cancer Institute, Penn State College of Medicine, Hershey, PA, USA; 45 Department of Biological and Environmental Sciences and Technologies (DiSTeBA), University of Salento, Lecce, Italy; 46 Department of Epidemiology and Preclinical Research, National Institute for Infectious Diseases Lazzaro Spallanzani, Istituto Ricovero Cura Carattere Scientifico (IRCCS), Rome, Italy; 47 Department of Immunobiology, Yale School of Medicine, New Haven, CT, USA; 48 Institute for Experimental Cancer Research in Pediatrics, Goethe University, Frankfurt, Germany; 49 INSERM, U866, Dijon, France; 50 Faculty of Medicine, University of Burgundy, Dijon, France; 51 Antiviral Immunity, Biotherapy and Vaccine Unit, Infection and Epidemiology Department, Institut Pasteur, Paris, France; 52 Department of Immunology, St Jude's Children's Research Hospital, Memphis, TN, USA; 53 Department of Functional Genomics and Cancer, Institut de Génétique et de Biologie Moléculaire et Cellulaire (IGBMC), Illkirch, France; 54 Department of Pathology, Anatomy and Cell Biology, Thomas Jefferson University, Philadelphia, PA, USA; 55 W Harry Feinstone Department of Molecular Microbiology and Immunology, Johns Hopkins University, Baltimore, MD, USA; 56 Institute of Molecular Life Sciences, University of Zurich, Zurich, Switzerland; 57 Laboratory of Cell Signaling, Graduate School of Pharmaceutical Sciences, University of Tokyo, Tokyo, Japan; 58 Department of Oncology-Pathology, Cancer Centrum Karolinska (CCK), Karolinska Institute, Stockholm, Sweden; 59 Medical Department for Hematology, Technical University of Munich, Munich, Germany; 60 Institute of Pharmacology, Medical Faculty, University of Bern, Bern, Switzerland; 61 Metabolomics and Cell Biology Platforms, Gustave Roussy Cancer Center, Villejuif, France; 62 Life Sciences Institute, University of Michigan, Ann Arbor, MI, USA; 63 Medical Molecular Biology Unit, Institute of Child Health, University College London (UCL), London, UK; 64 Medical Research Council Toxicology Unit, Leicester, UK; 65 Centre for Cancer Biology, University of South Australia, Adelaide, SA, Australia; 66 School of Medicine and School of Molecular and Biomedical Science, University of Adelaide, Adelaide, SA, Australia; 67 Departments of Drug Discovery and Biomedical Sciences and Biochemistry and Molecular Biology, Medical University of South Carolina, Charleston, SC, USA; 68 Center for Autophagy Research, University of Texas, Southwestern Medical Center, Dallas, TX, USA; 69 Howard Hughes Medical Institute (HHMI), Chevy Chase, MD, USA; 70 Division of Nephrology and Hypertension, Christian-Albrechts University, Kiel, Germany; 71 The Scripps Research Institute, La Jolla, CA, USA; 72 Sanford-Burnham Center for Neuroscience, Aging, and Stem Cell Research, La Jolla, CA, USA; 73 Salk Institute for Biological Studies, La Jolla, CA, USA; 74 University of California, San Diego (UCSD), San Diego, CA, USA; 75 Department of Biological Sciences, St. John's University, Queens, NY, USA; 76 Department of Biochemistry and Molecular Biology, Faculty of Medecine, Instituto Universitario de Oncología (IUOPA), University of Oviedo, Oviedo, Spain; 77 Unit of Clinical and Experimental Immunology, Humanitas Clinical and Research Center, Milan, Italy; 78 Institute of Molecular Biosciences, University of Graz, Graz, Austria; 79 Department of Therapeutic Research and Medicine Evaluation, Istituto Superiore di Sanita (ISS), Roma, Italy; 80 San Raffaele Institute, Sulmona, Italy; 81 Laboratory for Molecular Cancer Biology, Center for the Biology of Disease, Leuven, Belgium; 82 Laboratory for Molecular Cancer Biology, Center of Human Genetics, Leuven, Belgium; 83 Department of Genetics, The Smurfit Institute, Trinity College, Dublin, Ireland; 84 Department of Cell Biology, University of Geneva, Geneva, Switzerland; 85 Laboratory for Experiments Oncology and Radiobiology (LEXOR), Academic Medical Center (AMC), Amsterdam, The Netherlands; 86 Institute of Cancer Research, The Breakthrough Toby Robins Breast Cancer Research Centre, London, UK; 87 Department of Chemical Sciences and Technologies, University of Rome Tor Vergata, Rome, Italy; 88 Graduate School and Faculty of Medicine, University of Tokyo, Tokyo, Japan; 89 Department of Pathology, Stony Brook University, Stony Brook, NY, USA; 90 Cell Death Regulation Group, Bellvitge Biomedical Research Institute (IDIBELL), Barcelona, Spain; 91 Department of Pathology and Comprehensive Cancer Center, University of Michigan Medical School, Ann Arbor, MI, USA; 92 Department of Immunology, University of Washington, Seattle, WA, USA; 93 Institute of Molecular Biotechnology of the Austrian Academy of Sciences, Vienna, Austria; 94 Department of Hematology/Oncology, Feinberg School of Medicine, Northwestern University, Chicago, IL, USA; 95 Department of Morphology, Surgery and Experimental Medicine, Section of Pathology, Oncology and Experimental Biology and LTTA Center, University of Ferrara, Ferrara, Italy; 96 Department of Physiology and Medical Physics, Royal College of Surgeons, Dublin, Ireland; 97 Department of Biochemistry, La Trobe Institute of Molecular Science, La Trobe University, Melbourne, Australia; 98 Laboratory of Immunopathology, Instituto de Biología y Medicina Experimental (IBYME), Consejo Nacional de Investigaciones Científicas y Técnicas (CONICET), Buenos Aires, Argentina; 99 Department of Microbiology, Immunology and Cancer Biology, University of Virginia, Charlottesville, VA, USA; 100 Department Biomedical Sciences, University of Padova, Padova, Italy; 101 Research Institute for Medicines, Faculty of Pharmacy, University of Lisbon, Lisbon, Portugal; 102 Department of Medical Genetics, Cambridge Institute for Medical Research, University of Cambridge School of Clinical Medicine, Cambridge, UK; 103 Department of Microbiology, University of Würzburg; Würzburg, Germany; 104 Soochow Institute for Translational Medicine, Soochow University, Suzhou, China; 105 Institute of Pharmacology, University of Bern, Bern, Switzerland; 106 Departments of Biological Sciences and Chemistry, Columbia University, New York, NY, USA; 107 Department of Cell and Developmental Biology and Consortium for Mitochondrial Research, University College London (UCL), London, UK; 108 Cancer Research UK Beatson Institute, Glasgow, UK; 109 Institute of Cancer Sciences, University of Glasgow, Glasgow, UK; 110 Institute of Molecular Biology and Biotechnology, Foundation for Research and Technology-Hellas, Heraklion, Crete, Greece; 111 Department of Basic Sciences, Faculty of Medicine, University of Crete, Heraklion, Crete, Greece; 112 Osaka Medical Center for Cancer and Cardiovascular Diseases, Osaka, Japan; 113 Methusalem Program, Ghent University, Ghent, Belgium; 114 Division of Developmental Immunology, Biocenter, Medical University Innsbruck, Innsbruck, Austria; 115 Cancer Cell Biology Program, Spanish National Cancer Research Centre (CNIO), Madrid, Spain; 116 Centre for Cell Death, Cancer and Inflammation (CCCI), UCL Cancer Institute, University College London (UCL), London, UK; 117 Rutgers Cancer Institute of New Jersey, New Brunswick, NJ, USA; 118 Department of Pharmacology, University of Minnesota School of Medicine, Minneapolis, MN, USA; 119 Geriatric Research, Education and Clinical Center, VA Medical Center, Minneapolis, MN, USA; 120 Department of Cell Biology, Harvard Medical School, Boston, MA, USA; 121 Department of Biology, Queens College, Queens, NY, USA; 122 Graduate Center, City University of New York (CUNY), Queens, NY, USA; 123 Division of Toxicology, Institute of Environmental Medicine, Karolinska Institute, Stockholm, Sweden; 124 Faculty of Fundamental Medicine, Lomonosov Moscow State University, Moscow, Russia; 125 Pôle de Biologie, Hôpital Européen Georges Pompidou, AP-HP, Paris, France.

## Abstract

Cells exposed to extreme physicochemical or mechanical stimuli die in an uncontrollable manner, as a result of their immediate structural breakdown. Such an unavoidable variant of cellular demise is generally referred to as ‘accidental cell death' (ACD). In most settings, however, cell death is initiated by a genetically encoded apparatus, correlating with the fact that its course can be altered by pharmacologic or genetic interventions. ‘Regulated cell death' (RCD) can occur as part of physiologic programs or can be activated once adaptive responses to perturbations of the extracellular or intracellular microenvironment fail. The biochemical phenomena that accompany RCD may be harnessed to classify it into a few subtypes, which often (but not always) exhibit stereotyped morphologic features. Nonetheless, efficiently inhibiting the processes that are commonly thought to cause RCD, such as the activation of executioner caspases in the course of apoptosis, does not exert true cytoprotective effects in the mammalian system, but simply alters the kinetics of cellular demise as it shifts its morphologic and biochemical correlates. Conversely, *bona fide* cytoprotection can be achieved by inhibiting the transduction of lethal signals in the early phases of the process, when adaptive responses are still operational. Thus, the mechanisms that truly execute RCD may be less understood, less inhibitable and perhaps more homogeneous than previously thought. Here, the Nomenclature Committee on Cell Death formulates a set of recommendations to help scientists and researchers to discriminate between essential and accessory aspects of cell death.

Defining life and death is more problematic than one would guess. In 1838, the work of several scientists including Matthias Jakob Schleiden, Theodor Schwann and Rudolf Carl Virchow culminated in the so-called ‘cell theory', postulating that: (1) all living organisms are composed of one or more cells; (2) the cell is the basic unit of life; and (3) all cells arise from pre-existing, living cells.^1^ Only a few decades later (in 1885), Walter Flemming described for the first time some of the morphologic features that have been largely (but often inappropriately) used to define apoptosis throughout the past four decades.^2, 3, 4^

A corollary of the cell theory is that viruses do not constitute *bona fide* living organisms.^5^ However, the discovery that the giant *Acanthamoeba polyphaga* mimivirus can itself be infected by other viral species has casted doubts on this point.^6, 7, 8^ Thus, the features that underlie the distinction between a living and an inert entity remain a matter of debate. Along similar lines, defining the transition between an organism's life and death is complex, even when the organism under consideration is the basic unit of life, a cell. From a conceptual standpoint, cell death can obviously be defined as the permanent degeneration of vital cellular functions. Pragmatically speaking, however, the precise boundary between a reversible alteration in homeostasis and an irreversible loss of cellular activities appears to be virtually impossible to identify. To circumvent this issue, the Nomenclature Committee on Cell Death (NCCD) previously proposed three criteria for the identification of dead cells: (1) the permanent loss of the barrier function of the plasma membrane; (2) the breakdown of cells into discrete fragments, which are commonly referred to as apoptotic bodies; or (3) the engulfment of cells by professional phagocytes or other cells endowed with phagocytic activity.^9, 10, 11^

However, the fact that a cell is engulfed by another via phagocytosis does not imply that the cell-containing phagosome fuses with a lysosome and that the phagosomal cargo is degraded by lysosomal hydrolases.^12, 13, 14^ Indeed, it has been reported that engulfed cells can be released from phagosomes as they preserve their viability, at least under some circumstances.^15^ Thus, the NCCD recommends here to consider as *dead* only cells that either exhibit irreversible plasma membrane permeabilization or have undergone complete fragmentation. A compendium of techniques that can be used to quantify these two markers of end-stage cell death *in vitro* and *in vivo* goes beyond the scope of this review and can be found in several recent articles.^16, 17, 18, 19, 20, 21, 22, 23, 24, 25^

Importantly, cell death instances can be operationally classified into two broad, mutually exclusive categories: ‘accidental' and ‘regulated'. Accidental cell death (ACD) is caused by severe insults, including physical (e.g., elevated temperatures or high pressures), chemical (e.g., potent detergents or extreme variations in pH) and mechanical (e.g., shearing) stimuli, is virtually immediate and is insensitive to pharmacologic or genetic interventions of any kind. The NCCD thinks that this reflects the structural disassembly of cells exposed to very harsh physicochemical conditions, which does not involve a specific molecular machinery. Although ACD can occur *in vivo*, for instance as a result of burns or traumatic injuries, it cannot be prevented or modulated and hence does not constitute a direct target for therapeutic interventions.^23, 26, 27, 28^ Nonetheless, cells exposed to extreme physicochemical or mechanical insults die while releasing elevated amounts of damage-associated molecular patterns (DAMPs), that is, endogenous molecules with immunomodulatory (and sometimes cytotoxic) activity. Some DAMPs can indeed propagate an unwarranted cytotoxic response (directly or upon the involvement of innate immune effectors) that promotes the demise of local cells surviving the primary insult.^16, 19, 29, 30, 31^ Intercepting DAMPs or blocking DAMP-ignited signaling pathways may mediate beneficial effects in a wide array of diseases involving accidental (as well as regulated) instances of cell death.^19, 32^

At odds with its accidental counterpart, regulated cell death (RCD) involves a genetically encoded molecular machinery.^9, 33^ Thus, the course of RCD can be altered by means of pharmacologic and/or genetic interventions targeting the key components of such a machinery. Moreover, RCD often occurs in a relatively delayed manner and is initiated in the context of adaptive responses that (unsuccessfully) attempt to restore cellular homeostasis.^34, 35, 36, 37, 38^ Depending on the initiating stimulus, such responses can preferentially involve an organelle, such as the reticular unfolded protein response, or operate at a cell-wide level, such as macroautophagy (hereafter referred to as autophagy).^39, 40, 41, 42, 43, 44^ Thus, while ACD is completely unpreventable, RCD can be modulated (at least to some extent, see below) not only by inhibiting the transduction of lethal signals but also by improving the capacity of cells to mount adaptive responses to stress.^45, 46, 47, 48, 49, 50^ Importantly, RCD occurs not only as a consequence of microenvironmental perturbations but also in the context of (post-)embryonic development, tissue homeostasis and immune responses.^51, 52, 53, 54^ Such completely physiologic instances of RCD are generally referred to as ‘programmed cell death' (PCD) (Figure 1).^9, 33^

For the purpose of this discussion, it is useful to keep in mind the distinction that is currently made between the initiation of RCD and its execution. The term *execution* is generally used to indicate the ensemble of biochemical processes that truly cause the cellular demise. Conversely, *initiation* is commonly used to refer to the signal transduction events that activate executioner mechanisms. Thus, the activation of caspase-8 (CASP8) in the course of FAS ligand (FASL)-triggered apoptosis is widely considered as an initiator mechanism, whereas the consequent activation of caspase-3 (CASP3) is categorized as an executioner mechanism (see below).^51, 55, 56, 57^

Here, the NCCD formulates a set of recommendations to discriminate between *essential* and *accessory* aspects of RCD, that is, between those that etiologically mediate its occurrence and those that change its kinetics or morphologic and biochemical manifestations.

## Morphologic Aspects of Cell Death

The early classifications of cell death were purely morphologic, owing to obvious technical limitations.^18, 20^ In 1964, the American biologist Richard A Lockshin was the first to thoroughly describe the demise of intersegmental muscles in developing silk moths, a seminal contribution to the modern understanding of PCD.^58^ A few years later, in 1972, the Australian pathologist John F Kerr together with his Scottish colleagues Andrew H Wyllie and Alastair R Currie coined the term ‘apoptosis' (from the ancient Greek 
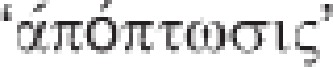
, meaning ‘falling off') to indicate a morphologically stereotyped form of cellular demise characterized by cytoplasmic shrinkage, chromatin condensation initiating at the nuclear membrane (marginalization) and then involving the whole nucleus (pyknosis), nuclear fragmentation (karyorrhexis), minimal alterations of other organelles and a peculiar ‘boiling-like' process (blebbing) culminating in the formation of a few discrete corpses that initially retain plasma membrane integrity (apoptotic bodies).^10, 11, 59^ Soon thereafter, the first ‘formal' classification of cell death differentiated between: (1) type I cell death (apoptosis), manifesting with the morphologic features described above; (2) type II cell death (autophagy), featuring an extensive vacuolization of the cytoplasm; and (3) type III cell death (necrosis), exhibiting neither apoptotic nor autophagic characteristics.^3, 60^ Such a ‘visual catalog' has dominated the field of cell death research for decades. Nonetheless, the NCCD views it as an oversimplification and considers it rather misleading, for several reasons.

First, when this classification was formulated, necrosis (as defined by morphologic features) was considered as a strict equivalent of ACD, whereas apoptosis (as defined by morphologic features) was viewed as the sole programmed subroutine of cell death.^11^ Along similar lines, apoptosis was misconceived as an immunologically silent, if not tolerogenic, cell death modality.^61, 62^ It is now clear that PCD does not always manifest with an apoptotic morphotype and does not necessarily fail to induce inflammatory or immune responses. For instance, the degradation of *Drosophila melanogaster* salivary glands and larval midgut relies on PCD manifesting with a type II morphology,^63, 64, 65, 66, 67, 68, 69, 70^ whereas the remodeling of bones at the growth plates is associated with type III-like features.^71^ Moreover, specific inducers are capable of promoting a variant of RCD that displays an apoptotic morphotype, yet is capable of activating adaptive immune responses.^72, 73^ These observations indicate that morphologic and functional aspects of cell death are not necessarily linked to each other.

Second, the negative morphologic definition of necrosis as a cell death modality that fails to exhibit apoptotic or autophagic features has been reconsidered.^22, 74^ Indeed, necrosis can manifest with a stereotyped panel of features, including a generalized swelling of the cytoplasm, which acquires a translucent aspect, and organelles (oncosis), as well as a peculiar alteration of chromatin (condensation into small and irregular patches) and the nuclear membrane (dilatation).^74^ The evolution of the morphologic characterization of necrosis reflects the relatively recent discovery that RCD can also manifest with a necrotic aspect (see below).

Third, the use of the term ‘autophagic cell death' has been a matter of intense debate.^75^ Such an expression was coined based on morphologic considerations (i.e., the appearance of autophagic vacuoles in the course of type II cell death) only, but it soon became misused to imply that the molecular machinery of autophagy would actively contribute to the cellular demise.^75^ The NCCD strongly recommends the use of the expression ‘autophagic cell death' from a functional perspective only, that is, to indicate a cell death subroutine that is limited or delayed by the pharmacologic or genetic inhibition of the autophagic machinery (see below).^9, 76^

Fourth, many instances of RCD present both apoptotic and necrotic traits.^10^ Moreover, several pharmacologic agents and genetic interventions designed to inhibit the execution of cell death often fail to do so when administered in a therapeutic (as opposed to prophylactic)^77^ manner, at least in the mammalian system, yet efficiently change its morphology.^78, 79, 80, 81, 82, 83, 84, 85, 86, 87, 88, 89, 90, 91^ This applies to *N*-benzyloxycarbonyl-Val-Ala-Asp(O-Me) fluoromethylketone (Z-VAD-fmk) and (3*S*)-5-(2,6-difluorophenoxy)-3-[[(2*S*)-3-methyl-1-oxo-2-[(2-quinolinylcarbonyl)amino]butyl]amino]-4-oxo-pentanoic acid hydrate (Q-VD-OPh), two broad-spectrum caspase inhibitors that have been widely investigated in the late 1990s as a means to mediate clinically relevant cytoprotection.^92, 93, 94, 95^ Z-VAD-fmk and Q-VD-OPh prevent the appearance of several morphologic markers of apoptosis, including nuclear pyknosis and blebbing (which rely on caspases),^96, 97^ yet fail to limit stimulus-dependent cell death if administered in therapeutic settings (i.e., after the cell death inducer).^85, 98, 99, 100^ Thus, caspase inhibition most often results in a shift from an overtly apoptotic to a mixed or necrotic cell death morphology.^81, 91^ Conversely, both necrostatin 1 (Nec-1), a highly specific small inhibitor of the enzymatic activity of receptor-interacting protein kinase 1 (RIPK1), and geldanamycin, which targets heat-shock protein 90 kDa alpha (cytosolic), class A member 1 (HSP90AA1), have been demonstrated to shift the necrotic morphotype of RCD induced by tumor necrosis factor (ligand) superfamily, member 10 (TNFSF10, best known as TNF-related apoptosis-inducing ligand, TRAIL) at slightly acidic pH to an apoptotic one.^101^ A similar morphologic shift has been observed in cells succumbing to tumor necrosis factor receptor superfamily, member 1A (TNFRSF1A, best known as tumor necrosis factor receptor 1, TNFR1) ligation in the presence of geldanamycin^102^ and in the absence of RIPK1 or one of its downstream targets, that is, receptor-interacting protein kinase 3 (RIPK3) and mixed lineage kinase domain-like (MLKL).^103, 104^ Also, cells exposed to DNA-alkylating agents in the presence of poly(ADP-ribose) polymerase 1 (PARP1) inhibitors die while manifesting an apoptotic, rather than a necrotic, morphology.^105, 106^ Conversely, the introduction of a non-cleavable PARP1 variant appears to convert the apoptotic phenotype of cells succumbing to FAS ligation into a necrotic one.^107^ Possibly, this reflects the ability of PARP1, an NAD^+^-dependent enzyme initially characterized for its role in DNA repair and the DNA damage response,^108, 109^ to provoke an abrupt decline in intracellular ATP levels (secondary to NAD^+^ depletion), hence blocking various morphologic manifestations of apoptosis.^110, 111, 112, 113, 114, 115^ Such a morphologic shift, however, does not appear to stem from the inhibition of caspases, because neither the catalytic functions nor the activation of these proteases require ATP (which should not be confounded with deoxy-ATP, see below).^116, 117, 118, 119^

In summary, the morphologic manifestations of cell death can easily be altered in the absence of *bona fide* cytoprotection, casting doubts on the actual value of morphology-based classifications of cell death.^9^

## Biochemical Manifestations of Cell Death

In 2012, the NCCD proposed to abandon the morphologic catalog of cell death instances in favor of a new classification based on quantifiable biochemical parameters.^9^ In substitution, the NCCD identified the main molecular events associated with specific cell death subroutines as well as the pharmacologic and/or genetic interventions that may be used to discriminate between various instances of cell death in experimental settings, *in vitro* and *in vivo*.^9^

Since then, our comprehension of specific RCD modalities has progressed significantly. Thus, while no paradigm-breaking discoveries have been made on the regulation and execution of caspase-dependent RCD instances (which most often display an apoptotic morphology), profound insights have been obtained into the mechanisms underlying cases of RCD that do not depend on caspases and generally manifest with necrotic features.^33, 74, 120, 121^ This notion began to emerge in the late 1980s,^122^ but became widely accepted only two decades later, owing to the milestone discoveries of Peter Vandenabeele,^123, 124, 125, 126, 127^ Jurg Tschopp^128^ and Junying Yuan,^129, 130, 131^ and to the characterization of the key role played by peptidylprolyl isomerase F (PPIF, best known as cyclophilin D, CYPD) in necrotic instances of RCD.^132, 133, 134, 135^ The identification of a genetically encoded machinery that promotes RCD with necrotic features generated an intense wave of investigation that has not yet come to an end.^33, 74, 120, 121^

From a biochemical standpoint, apoptosis is defined as a caspase-dependent variant of RCD.^9, 51^ Other events commonly associated with apoptosis, such as the exposure of phosphatidylserine on the outer leaflet of the plasma membrane, are indeed less universal and more context-dependent^136, 137, 138, 139^ than previously thought.^140^ Apoptosis can be initiated by intracellular (intrinsic) or extracellular (extrinsic) stimuli. Intrinsic apoptosis critically relies on mitochondrial outer membrane permeabilization (MOMP), a process that results in the holocytochrome *c* (CYT*C*)-, deoxy-ATP- and apoptotic peptidase-activating factor 1 (APAF1)-dependent activation of caspase-9 (CASP9) and CASP3.^117, 141, 142, 143, 144^ MOMP obligatorily requires either of two Bcl-2 family members, namely, B-cell CLL/lymphoma 2 (BCL2)-associated X protein (BAX) and BCL2-antagonist/killer 1 (BAK1), whose pore-forming activity is inhibited (both directly and via indirect circuitries) by other components of the family, including BCL2 itself, BCL2-like 1 (BCL2L1, best known as BCL-X_L_) and myeloid cell leukemia 1 (MCL1).^48, 145, 146, 147, 148^ Importantly, the physical and functional interactions between pro- and antiapoptotic multidomain BCL2-like proteins are under the control of small components of the family known as BH3-only proteins, including (but not limited to) BCL2 binding component 3 (BBC3, best known as PUMA), BCL2-like 11 (BCL2L11, best known as BIM) and BH3-interacting domain death agonist (BID).^149, 150, 151^

Extrinsic apoptosis proceeds along with the activation of a CASP8/CASP3 signal transduction axis that in some cell types (including hepatocytes, pancreatic *β* cells and multiple neoplastic cells) also involves MOMP, owing to the CASP8-dependent activation of BID.^152, 153, 154, 155, 156, 157^ Whether the apoptotic response to extracellular cues requires MOMP or not reportedly depends on the expression levels of X-linked inhibitor of apoptosis (XIAP),^158, 159^ an ubiquitin ligase with multipronged cytoprotective functions.^160, 161^ High amounts of XIAP prevent indeed the direct activation of CASP3 by CASP8, a block that can be circumvented by the release of diablo, IAP-binding mitochondrial protein (DIABLO, best known as second mitochondria-derived activator of caspases, SMAC) and other XIAP inhibitors into the cytosol following MOMP.^158, 159, 162, 163, 164^

It recently became clear that the main players in the RCD subroutine commonly referred to as necroptosis, which we previously defined as a caspase-independent, RIPK1- and RIPK3-dependent lethal signaling pathway initiated by death receptors,^9, 165^ include not only RIPK1 and RIPK3, as initially thought,^120, 121, 131, 166, 167, 168, 169^ but also MLKL.^104, 170, 171, 172, 173, 174, 175, 176, 177, 178, 179^ The kinase activity of RIPK1 is required for necroptosis as induced by multiple stimuli, including death receptor ligation in the presence of caspase inhibitors.^128, 129, 130, 131, 169, 180^ Conversely, catalytically inactive and Nec-1-bound RIPK1 inhibits the necroptotic response of CASP8-incompetent cells to Toll-like receptor (TLR) agonists or type I interferons, which relies not only on RIPK3 but also on TLR adaptor molecule 1 (TICAM1, best known as TRIF).^181, 182, 183, 184^ In the absence of RIPK1, TLR agonists and type I interferons trigger necroptosis even in caspase-competent cells,^182, 183^ suggesting that RIPK1 can inhibit necroptotic RCD as induced by these stimuli at two distinct levels. Furthermore, RIPK1 tonically suppresses CASP8 and RIPK3 activation in developmental scenarios independent of its kinase activity.^183, 185, 186^ This explains why *Ripk1*^*−/−*^ mice fail to survive to adulthood even in the absence of Fas (TNFRSF6)-associated via death domain (*Fadd*), which is required for CASP8 activation by extracellular cues, but mature normally in the absence of both *Ripk3* and *Fadd* or *Casp8*.^183^ Other instances of necroptosis, such as those promoted by Z-DNA-binding protein 1 (ZBP1) in response to viral infection, appear to proceed independently of RIPK1.^187^ Upon phosphorylation by RIPK3, MLKL has a critical, non-redundant role in necroptosis.^177, 179^ Phosphorylated MLKL forms indeed oligomers that translocate to cellular membranes (including the plasma membrane) and bind specific phospholipids, resulting in the loss of barrier function.^170, 171, 174, 175, 188^

Recent data argue against an essential role for mitochondria in necroptosis. Indeed, parkin RBR E3 ubiquitin protein ligase (PARK2)-overexpressing cells depleted of the vast majority of mitochondria upon the induction of mitophagy (by means of a mitochondrial uncoupler) become resistant to inducers of MOMP-dependent RCD, but remain sensitive to TNFR1 ligation in the presence of Z-VAD-fmk (a conventional trigger of necroptosis).^189^ Moreover, contrary to initial beliefs,^190^ the lethal activity of RIPK3 is not influenced by the absence of phosphoglycerate mutase family member 5 (PGAM5) and dynamin 1 like (DNM1L, best known as dynamin-related protein 1, DRP1).^104, 172, 176^ Based on these results, the NCCD proposes here to redefine necroptosis as an RCD modality that critically depends on MLKL and on the kinase activity of RIPK1 (in some settings) and RIPK3. Of note, both RIPK1 and RIPK3 have been shown to regulate caspase activation, at least under some circumstances.^186, 191, 192, 193^ Taken together, these observations suggest that the signal transduction cascades responsible for the initiation of apoptosis and necroptosis are highly interconnected.

Necroptosis is actively inhibited by a supramolecular complex containing CASP8, FADD and the long isoform of CASP8 and FADD-like apoptosis regulator (CFLAR, best known as cellular FLICE inhibitor protein, c-FLIP),^194, 195, 196, 197^ three key components of caspase-dependent RCD initiated by death receptors.^198, 199, 200, 201, 202, 203^ Taken together with the notion that the absence of either *Ripk3*, *Casp8* or *Fadd* fails to rescue *Ripk1*^*−/−*^ mice from neonatal lethality,^180, 185^ these results pointed to the existence of a switch mechanism that regulates cell fate upon TNFR1 ligation.^204, 205^ Intriguingly, such switch may not operate in all cell types, as demonstrated by the fact that *Ripk1*^*−/−*^ intestinal epithelial cells are fully rescued by the concomitant absence of *Casp8* (Peter Vandenabeele, personal communication).

Recent data obtained with genetically engineered RIPK1 and RIPK3 variants indicate that the catalytic pathways activated in response to death receptor ligation depend on the availability of CASP8, FADD and MLKL.^206^ In comparatively more physiologic conditions, however, the fate of cells exposed to death receptor ligands may be determined by the activation kinetics of mitogen-activated protein kinase kinase kinase 7 (MAP3K7, best known as TGF*β*-activated kinase 1, TAK1),^192, 193, 207^ which normally initiates a cytoprotective response centered around the transcription factor NF-κB and autophagy,^208, 209, 210, 211, 212^ or by the availability of baculoviral IAP repeat containing (BIRC) family members,^192, 193^ ubiquitin ligases with a central role in TNFR1 signaling.^213^ In line with this notion, cells treated with a SMAC mimetic (resulting in the depletion of BIRC2 and BIRC3) or a chemical TAK1 inhibitor (NP-009245) reportedly respond to TNFR1 ligation by activating caspases in a RIPK1-dependent manner.^192^ Taken together, these observations indicate that death receptors generate a lethal stimulus that can be propagated along several signal transduction cascades. Thus, caution should be used in evaluating necroptotic instances of cell death based on their sensitivity to Nec-1 only.

Another variant of RCD that often, although not always, manifests with a necrotic morphotype critically relies on CYPD.^214^ At present, CYPD is the sole genetically confirmed component of the permeability transition pore complex (PTPC) in the mammalian system.^132, 133, 135, 215, 216, 217^ The term PTPC generally refers to a supramolecular complex operating at the junctions between the inner and outer mitochondrial membrane to cause the so-called ‘mitochondrial permeability transition' (MPT), an abrupt increase in the permeability of the inner mitochondrial membrane to small solutes triggered by cytosolic Ca^2+^ overload or oxidative stress.^214, 218, 219, 220, 221^ Unlike MOMP,^205, 222, 223, 224^ MPT seals the cell fate independently of caspase activation.^133, 225, 226^ Nonetheless, MPT-driven RCD can manifest with (at least some) morphologic features associated with apoptosis,^10, 227, 228^ corroborating the limited informative value of cell death classifications solely based on morphology. The NCCD recommends the use of the term ‘MPT-driven RCD' for instances of cell death whose course can be influenced with the genetic or pharmacologic inhibition of CYPD or other components of the PTPC. Of note, CYPD surely does not constitute the long-sought pore-forming unit of the PTPC, which most likely involves subunits of the so-called ‘ATP synthasome', the supramolecular complex that imports ADP and inorganic phosphate into the mitochondrial matrix, catalyzes ATP synthesis and exports ATP back to the mitochondrial intermembrane space (from where it can easily reach the cytosol).^229, 230, 231, 232, 233, 234^ Perhaps, the central role of CYPD in MPT-driven RCD reflects its ability to control the Ca^2+^-buffering capacity of the mitochondrial network.^235, 236^ This hypothesis has not yet been formally addressed.

Two forms of RCD other than necroptosis and MPT-driven RCD have recently attracted attention as potential targets for the development of cytoprotective interventions, namely ‘parthanatos' and ‘ferroptosis'.^33^ The main molecular features of parthanatos are the hyperactivation of PARP1 and the release of apoptosis-inducing factor, mitochondrion-associated, 1 (AIFM1) from the mitochondria.^237, 238, 239, 240^ Interestingly, although TNFR1-driven necroptosis and parthanatos have been suggested to constitute completely independent RCD subroutines,^241^ this issue remains a matter of debate.^101, 242^ Possibly, such a controversy originates from the ability of some insults to simultaneously trigger necroptosis and parthanatos, at least in some model systems.^243^ Ferroptosis has been defined as an iron-dependent form of RCD under the control of glutathione peroxidase 4 (GPX4).^244, 245, 246, 247^ Both the pharmacologic and genetic inhibition of CYPD fail to prevent ferroptosis as triggered by erastin, a small molecule that is selectively lethal for cancer cells expressing oncogenic variants of Harvey rat sarcoma viral oncogene homolog (HRAS).^246, 248^ This suggests that ferroptosis and MPT-driven RCD constitute independent variants of RCD. Of note, erastin inhibits system x_C_^−^, an heterodimeric antiporter of the plasma membrane that normally exchanges intracellular glutamate for extracellular cysteine, resulting in glutathione depletion and iron-dependent accumulation of reactive oxygen species.^247^ A similar cascade of events contributes to (but is not the sole etiological determinant of) the death of neurons exposed to glutamate. This necrotic instance of RCD has previously been referred to as oxytosis.^121, 249^ Besides inhibiting system x_C_^−^, glutamate can trigger MPT-driven RCD upon the hyperactivation of ionotropic receptors, a neurotoxic process commonly known as excitotoxicity.^250, 251^

Caspase-unrelated variants of RCD include ‘autophagic cell death', which (among other processes) is biochemically associated with the lipidation of microtubule-associated protein 1 light chain 3 (MAP1LC3, best known as LC3) and the degradation of sequestosome 1 (SQSTM1, best known as p62).^76^ The NCCD recommends using this term only for RCD instances that can be influenced by the pharmacologic or genetic interventions targeting at least two distinct components of the molecular machinery for autophagy.^9, 76^ While autophagy accompanies RCD in a vast number of pathophysiologic settings,^36, 50, 252^ it truly contributes to the cellular demise only in a few of them.^69, 70, 76, 253, 254, 255, 256, 257, 258, 259^ Beth Levine's laboratory has recently discovered a *bona fide* instance of autophagic cell death that relies on the plasma membrane Na^+^/K^+^-ATPase, and dubbed it ‘autosis'.^255^ Of note, autosis occurs not only *in vitro*, in cells exposed to cell permeant autophagy-inducing peptides, but also *in vivo*, in the brain of rats subjected to an ischemic insult.^255^ It remains to be determined whether all cases of autophagic cell death require the Na^+^/K^+^-ATPase or not. If so, the terms ‘autosis' and ‘autophagic cell death' would be synonymous. If not, autosis would constitute a special instance of autophagic cell death.^260^

Importantly, a growing body of evidence indicates that the pharmacologic or genetic inhibition of the processes that are commonly considered as essential for cell death execution often does not avoid the demise of mammalian cells, but rather alters its kinetics and biochemical (and morphologic) manifestations. Thus, in many experimental paradigms (*in vitro* and *in vivo*), Z-VAD-fmk and more specific CASP3 inhibitors administered as therapeutic (as opposed to prophylactic)^77^ interventions fail to significantly limit primary RCD, in spite of the fact that they efficiently limit caspase activation.^261, 262, 263, 264, 265^ In some of these scenarios, RCD overtly manifests with alternative biochemical processes, including RIPK1, RIPK3 or PARP1 activation, and (at least in part) can be influenced by agents that interfere with these pathways, including Nec-1, 3-aminobenzamide (a PARP1-targeting agent) and necrosulfonamide (an inhibitor of human MLKL).^261, 263, 264^ However, the proportion of cells *eventually* succumbing to RCD does not change. The depletion of BIRC2/BIRC3, RIPK1, RIPK3 or MLKL, and the administration of NP-009245, Nec-1 or geldanamycin (which indirectly destabilizes RIPK1)^266^ reportedly changes the kinetics of necroptosis and its biochemical profile, that is, it allows for caspase activation, yet fails to block the cellular demise.^101, 103, 104, 192, 267^ Along similar lines, 3-aminobenzamide and 4-amino-1,8-naphthalimide (another PARP1 inhibitor) can convert the cytotoxic response to alkylating DNA damage or TNFR1 ligation from a caspase-independent one to apoptosis, manifesting with caspase activation.^105, 106, 107^

These observations indicate that, similar to their morphologic counterparts, the biochemical manifestations of cell death can be altered in the absence of efficient cytoprotection.

## RCD and Stress Adaptation

**Figure 2:**
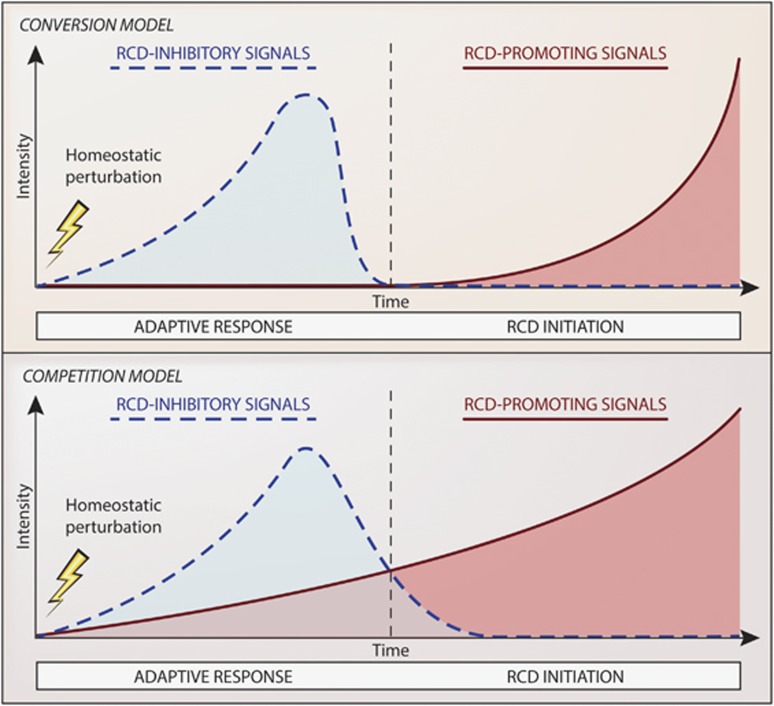
Regulated cell death and adaptive stress responses. Regulated cell death (RCD) is often initiated in the context of unsuccessful responses to perturbations of intracellular or extracellular homeostasis. Two mutually exclusive models can be put forward to explain how failing responses to stress initiate RCD (which in many instances constitutes a means to preserve the homeostasis of the whole organism or colony). First, according to a ‘conversion model', RCD-inhibitory signals simply cease at some stage of the adaptive response and are substituted by RCD-promoting ones. Second, a ‘competition model' postulates that RCD-inhibitory and -promoting signals coexist and counteract each other starting from the very detection of microenvironmental alterations, and at some stage the latter predominate over the former. Circumstantial evidence favors the ‘competition model' in a majority of experimental scenarios

Cells subjected to perturbations of intracellular or extracellular homeostasis almost invariably mount a tightly coordinated response aimed at (1) the removal of the initiating stimulus (when possible), (2) the repair of molecular and/or organelle damage, and (3) eventually, the re-establishment of physiologic conditions.^34, 35, 36, 37, 38, 42, 57, 268^ When these objectives cannot be attained, cells generally undergo RCD as a means to preserve the homeostasis of the whole organism (or colony, in the case of yeast cells). Two mutually exclusive models can be put forward to explain how adaptive stress responses promote RCD when unsuccessful. First, a ‘conversion model' postulates that RCD-inhibitory signals cease at some stage of the adaptive response and are replaced by RCD-promoting ones. Second, a ‘competition model' hypothesizes that RCD-inhibitory and -promoting signals coexist and counteract with each other starting from the detection of microenvironmental alterations, but at some stage the latter predominate over the former (Figure 2). Although data formally favoring one of these models over the other are lacking, circumstantial evidence suggests that RCD-promoting signals are activated when RCD-inhibitory mechanisms are still operational.^36, 159, 252, 269, 270, 271, 272, 273^

**Figure 3:**
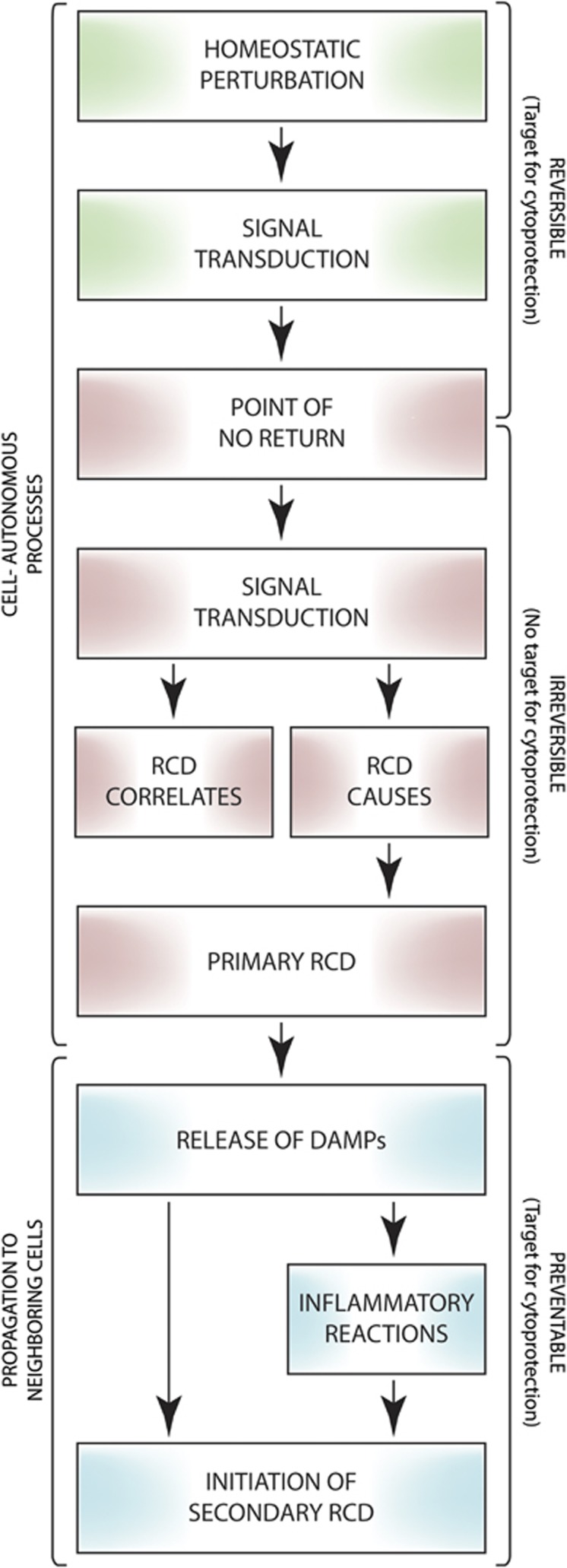
Initiation, execution and propagation of regulated cell death. The term ‘execution' has largely been used to indicate the processes that (were thought to) mediate regulated cell death (RCD), such as the massive activation of CASP3 in the course of apoptosis. Conversely, the word ‘initiation' has generally been used to refer to the signal transduction events that trigger executioner mechanisms, such as the activation of CASP8 or CASP9, both of which normally impinge on CASP3. Upon an attentive re-evaluation of the available literature, the NCCD recommends caution in attributing a specific process a *bona fide* causative value in the execution of cell death. In addition, the NCCD proposes to use the term ‘initiation' with a pragmatic connotation, that is, to indicate the steps in the cascades of events leading to RCD that are truly reversible, and the term ‘propagation' to indicate the processes that link primary RCD to the insult-independent initiation of a secondary wave of RCD, that is, the release of cytotoxic and proinflammatory factors, including damage-associated molecular patterns (DAMPs), by dying cells and their consequences. Based on this conceptual construction, only pharmacologic and genetic interventions that target the initiation phase exert *bona fide* cytoprotective effects, that is, truly inhibit primary RCD rather than just delaying its course or changing its morphologic or biochemical correlates. Robust cytoprotection can also be achieved *in vivo* by the administration of anti-inflammatory agents and by measures that block DAMPs or their receptors. These maneuvers, however, appear to be efficient as they prevent the propagation of primary RCD or the initiation of secondary RCD

Based on this conceptual construction, the NCCD recommends to use the term ‘initiation' to indicate the RCD-causing events that are reversible, that is, that do not irrevocably commit cells to die as they occur when adaptive responses are still operational. In addition, we encourage the use of the expression ‘execution' for referring to the processes that *irreversibly and causally* seal the cell fate, and the term ‘propagation' to indicate the processes that link primary (stimulus-dependent) RCD to the stimulus-independent initiation of a secondary RCD wave, including the release of DAMPs and the consequent inflammatory response (Figure 3). The blockage of RCD-initiating mechanisms by either pharmacologic or genetic means has been associated with consistent degrees of cytoprotection in rodent models representing various human diseases linked to unwarranted cell death. For instance, this applies to the whole-body ablation of *Ppif* (the CYPD-coding gene),^132, 133, 134, 274^ *Ripk3*^167, 176, 274, 275, 276, 277, 278^ and *Mlkl*,^176, 177^ as well as to the administration of chemical CYPD inhibitors (i.e., cyclosporin A and sanglifehrin A)^274^ and Nec-1.^274, 277^ Conversely, pharmacologic and genetic interventions expected to interrupt RCD at late steps of the process (when cells are commonly considered as committed to die) generally fail to confer significant long-term cytoprotection in mammalian models, casting doubts on the actual etiological value of these steps for RCD. Thus, *Casp3*^*−/−*^, *Casp9*^*−/−*^ and *Apaf1*^*−/−*^ mice display a consistent hyperplasia of the central nervous system associated with reduced amounts of PCD in specific cerebral areas, resulting in embryonic or perinatal lethality.^279, 280, 281, 282, 283, 284^ However, the neuronal phenotype of *Casp3*^*−/−*^ mice does not develop in all genetic backgrounds,^89, 285, 286^ and the penetrance of the perinatal lethality associated with the *Apaf1*^*−/−*^ genotype is incomplete (as some animals survive through 10 months of age).^88, 287^ Moreover, the developmental death of interdigital cells (which generally manifests with biochemical correlates of apoptosis) occurs close to normally (allowing for normal morphogenesis) in mice bearing a homozygous loss-of-function mutation in *Apaf1*, and in mice exposed to broad-spectrum caspase inhibitors.^80^ In these settings, however, the demise of interdigital cells cannot be detected by the terminal-deoxynucleotidyl-mediated dUTP nick end-labeling (TUNEL) assay, measuring caspase-dependent DNA fragmentation.^80^ Conversely, the simultaneous ablation of *Bax* and *Bak1*^288^ or *Bcl2l11* and *Bmf* (encoding two BH3-only proteins involved in MOMP initiation)^289, 290, 291^ truly prevents the programmed demise of several cell types, causing their persistence throughout adult life.^292, 293^ These observations are compatible with the hypothesis that the phenotype associated with some defects in the molecular cascades linking MOMP to caspase activation originates from a delay, rather than from *bona fide* inhibition, of PCD. Moreover they suggest that RCD, be it programmed or caused by microenvironmental perturbations, can only be avoided by interventions that target upstream steps of the process.

Caution should also be taken in inferring the actual etiological value of caspases in RCD based on the therapeutic administration of Z-VAD-fmk or other broad-spectrum caspase inhibitors. Caspase blockers have indeed been associated with (at least some degree of) cytoprotection in rodent models of various human diseases linked to the excessive loss of parenchymal cells. These pathologies include, but are not limited to, neurodegenerative disorders,^294, 295, 296, 297, 298^ traumatic events^299, 300^ and ischemia/reperfusion injuries of the central nervous system, heart and kidney.^301, 302, 303, 304^ Nonetheless, Z-VAD-fmk and similar compounds inhibit not only several caspases but also a wide panel of non-caspase proteases that participate in the initiation of RCD, such as calpains.^305, 306^ Moreover, CASP3, caspase-6 (CASP6) and caspase-7 (CASP7) (i.e., the putative executioners of apoptosis) have been involved in feedforward circuitries that amplify lethal cues leading to MOMP,^307, 308, 309, 310^ implying that their inhibition may also counteract the initiation of RCD. Finally, in models of this type it is difficult to discriminate between the primary wave of RCD (promoted by experimental interventions) and the delayed, secondary demise of parenchymal cells caused by DAMPs (directly or upon the establishment of inflammation).^73, 311, 312, 313^ The cytoprotective effects that Z-VAD-fmk-like chemicals exert in similar scenarios, which are most reliably evaluated by histological determinations or functional tests, might therefore reflect their ability to block the initiation of DAMP- and inflammation-driven secondary RCD rather than the execution of stimulus-induced, primary RCD. In line with this notion, consistent cytoprotection has also been achieved *in vivo* by means of anti-inflammatory agents, even when these compounds do not directly influence RCD.^314, 315, 316, 317^ Taken together, these observations reinforce the notion that caspases may not mediate RCD but simply accelerate its course, at least in the mammalian system.

Apparently at odds with the role of PARP1 in the execution of parthanatos, both the *Parp1*^*−/−*^ genotype and the administration of (relatively unselective) PARP inhibitors have been associated with *bona fide* cytoprotection in rodent models of ischemia/reperfusion injury and retinal degeneration.^318, 319, 320, 321^ These observations suggest that PARP1 and/or other members of the PARP family also participate in the initiation of RCD. Alternatively, the inhibition of PARP1 may limit the release of DAMPs or the consequent inflammatory response, at least in some pathophysiologic settings. Further experiments are required to clarify these possibilities.

## Concluding Remarks

As discussed above, the processes that until now were thought to mediate RCD most often do not causally underpin the cellular demise but represent biochemical manifestations of it. A growing body of data indicates indeed that the *bona fide* executioners of RCD, that is, the processes that directly drive cells across the boundary between life and death are less characterized, less inhibitable and perhaps more homogeneous than previously thought.^322^ In line with this theoretical construction, here the NCDD proposes to use the term ‘initiation' to refer to all the steps in the RCD cascade that are reversible, that is, which occur before cells make an irrevocable commitment to die. An attentive reinterpretation of the literature suggests that actual cytoprotection can only be achieved with pharmacologic or genetic interventions that inhibit or outcompete lethal signals at this stage, when adaptive responses to stress are still operational. Interestingly, some cells manifesting biochemical and morphologic features associated with late-stage RCD (including partial MOMP, caspase activation and blebbing) appear to recover (upon removal of the RCD-initiating insult) and replicate, a process that has been dubbed ‘anastasis' (from the ancient Greek ‘ανστασις', meaning ‘raising to life').^323, 324^ This suggests that the actual point of no return in the signal-transduction cascades leading to RCD may exhibit at least some degree of context dependency.

In the vast majority of scientific reports, RCD is measured *in vitro* 24–96 h after stimulation, whereas the most reliable assessment of RCD *in vivo* is based upon histologic determinations or functional tests performed days, if not weeks or months, after such experimental interventions. In the former scenario, investigators can collect valuable kinetic data but are unable to estimate the true long-term survival of cells exposed to perturbations of homeostasis. In the second scenario, it is difficult to discriminate between the interruption of primary RCD and the inhibition of DAMP-driven inflammatory reactions and secondary RCD. This may have profound mechanistic and therapeutic implications. Indeed, retarding the demise of a cell that has already committed to die in an irreversible manner, and the biochemical manifestations of such death, may have limited cytoprotective effects for the cell in question, but may impact on the emission of DAMPs and hence significantly influence RCD propagation. Thus, considerable degrees of cytoprotection might be attained by means of agents that interrupt lethal cues at (or before) RCD initiation (when adaptive stress responses are still functional) combined with strategies that inhibit propagation (e.g., chemicals that favor RCD instances associated with a limited release of DAMPs, DAMP-neutralizing measures, anti-inflammatory agents). The superior beneficial effects of cyclosporin A may indeed stem from its ability to inhibit MPT-driven RCD and simultaneously exert a robust anti-inflammatory activity.^325, 326^

**Figure 4:**
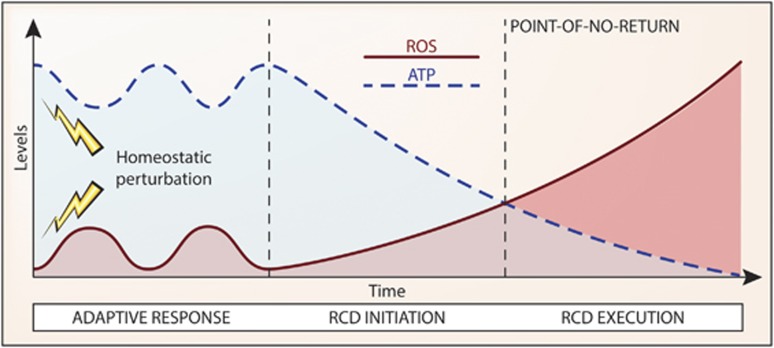
Declining ATP levels and redox alterations as a potential cause of regulated cell death. A growing amount of evidence indicates that the pharmacologic or genetic inhibition of the mechanisms that are commonly regarded as the executioners of regulated cell death (RCD) changes the kinetics of the process while altering its morphologic and biochemical manifestations, but fails to mediate *bona fide* long-term cytoprotection. It is therefore difficult to evaluate the actual causes that push cells beyond the point-of-no-return between life and death, especially as it remains to be formally demonstrated where the frontier between reversible alterations of homeostasis and the irreversible degeneration of cellular functions stands. ATP is required for a wide panel of vital activities, including the maintenance of the ionic equilibrium across the plasma membrane, implying that the drop of ATP concentrations below a specific threshold level may irremediably compromise the ability of cells to maintain structural integrity (which is the most reliable marker of cell death currently available). Along similar lines, variations in the oxidative potential of the intracellular milieu not only inhibit several enzymatic activities, including mitochondrial ATP synthesis, but also cause direct structural damage to organelles and membranes. We therefore hypothesize that declining ATP levels and a compromised redox homeostasis may constitute common causes of cell death in many RCD models. ROS, reactive oxygen species

If caspases and other enzymes commonly thought to mediate RCD in mammalian cells only underpin its manifestations, what are the true causes of cell death? Although the concentration of ATP is preserved (or even increases to some extent) in the course of adaptive stress responses,^247, 327^ circumstantial evidence points to dropping ATP levels, which at some point abolish the activity of all ATP-dependent enzymes (including various transporters that maintain ionic balance at the plasma membrane) and a compromised redox balance (which inactivates various enzymes and causes oxidative molecular damage to organelles and membranes) as central players in the execution of RCD (Figure 4).^328^ Alternatively, one or more hitherto uncharacterized mechanism(s) may causally underpin RCD in all its manifestations. Further experiments are required to explore these possibilities.

At odds with mammalian models, *Caenorhabditis elegans* and *D. melanogaster* are truly protected by Z-VAD-fmk and by the genetic inhibition of caspase orthologs and other proteins involved in the postmitochondrial phase of apoptosis.^329, 330, 331, 332, 333, 334, 335^ This may indicate that the signal transduction cascades underlying RCD are interconnected in a different manner in mammals and non-mammalian organisms. Alternatively, the actual requirement of caspases for (at least some instances of) RCD might have been concealed by the evolutionary expansion of the caspase family. Both the human and murine genome encode indeed 14 distinct caspases,^336, 337^ and it seems unlikely that Z-VAD-fmk and other pharmacologic or genetic interventions may simultaneously inhibit all of them in an efficient manner.

Until these uncertainties have been resolved, the NCCD recommends that investigators focus on essential aspects of cell death; first of all its actual occurrence. It appears indeed that measuring the functional status or subcellular localization of RCD-relevant proteins including (but not limited to) caspases, RIPK1, RIPK3, MLKL, CYPD, PARP1 and GPX4, can provide insights into the mechanisms that accompany (and regulate the kinetics of) cellular demise, but not into those that truly push cells beyond the point-of-no-return separating life and death. Precisely defining where this border stands from a bioenergetic and metabolic perspective may facilitate the development of novel and efficient cytoprotective agents for clinical use.

## Figures and Tables

**Figure 1:**
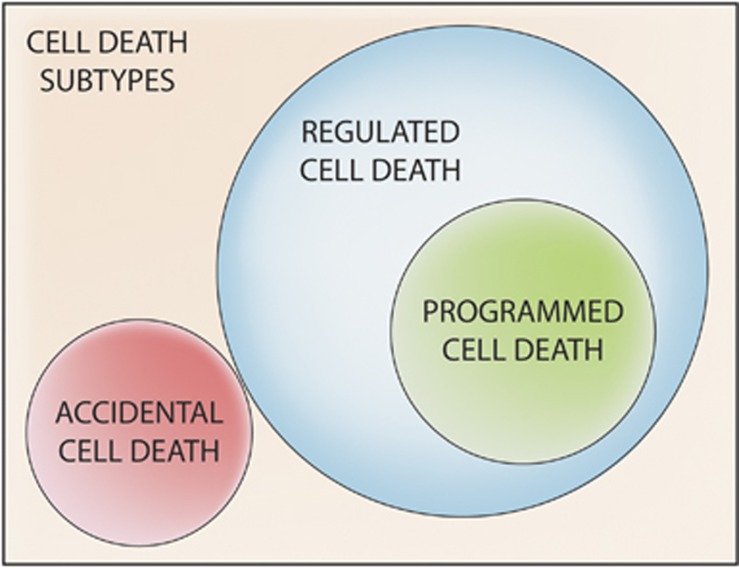
Types of cell death. Cells exposed to extreme physical, chemical or mechanical stimuli succumb in a completely uncontrollable manner, reflecting the immediate loss of structural integrity. We refer to such instances of cellular demise with the term ‘accidental cell death' (ACD). Alternatively, cell death can be initiated by a genetically encoded machinery. The course of such ‘regulated cell death' (RCD) variants can be influenced, at least to some extent, by specific pharmacologic or genetic interventions. The term ‘programmed cell death' (PCD) is used to indicate RCD instances that occur as part of a developmental program or to preserve physiologic adult tissue homeostasis

## Abbreviations

ACD: accidental cell death
APAF1: apoptotic peptidase-activating factor 1
BAX: BCL2-associated X protein
BAK1: BCL2-antagonist/killer 1
BCL2: B-cell CLL/lymphoma 2
BID: BH3-interacting domain death agonist
BIRC: baculoviral IAP repeat containing
CASP3: caspase-3
CASP8: caspase-8
CASP9: caspase-9
CYPD: cyclophilin D
DAMP: damage-associated molecular pattern
FADD: Fas (TNFRSF6)-associated via death domain
GPX4: glutathione peroxidase 4
MLKL: mixed lineage kinase domain-like
MOMP: mitochondrial outer membrane permeabilization
MPT: mitochondrial permeability transition
NCCD: Nomenclature Committee on Cell Death
Nec-1: necrostatin 1
PARP1: poly(ADP-ribose) polymerase 1
PCD: programmed cell death
PPIF: peptidylprolyl isomerase F
PTPC: permeability transition pore complex
Q-VD-OPh: (3*S*)-5-(2,6-difluorophenoxy)-3-[[(2*S*)-3-methyl-1-oxo-2-[(2-quinolinylcarbonyl)amino]butyl]amino]-4-oxo-pentanoic acid hydrate
RCD: regulated cell death
RIPK1: receptor-interacting protein kinase 1
RIPK3: receptor-interacting protein kinase 3
SMAC: second mitochondria-derived activator of caspases
TAK1: TGF*β*-activated kinase 1
TLR: Toll-like receptor
TNFR1: tumor necrosis factor receptor 1
XIAP: X-linked inhibitor of apoptosis
Z-VAD-fmk: *N*-benzyloxycarbonyl-Val-Ala-Asp(O-Me) fluoromethylketone
